# The toxic effect of titanium dioxide nanoparticles on rat submandibular salivary glands and the protective role of vitamin E

**DOI:** 10.1186/s12903-025-06631-w

**Published:** 2025-08-20

**Authors:** Mariam Aboayana, Yasmine M. Tolba

**Affiliations:** Department of Oral Biology, Faculty of Dentistry, Alexandria University, Elmassalah, Alexandria Egypt

**Keywords:** Nanotechnology, TiO_2_, Nanoparticles, Vitamin E, Submandibular glands

## Abstract

**Background:**

To assess the impact of titanium dioxide nanoparticles (TiO_2_NPs) on submandibular salivary glands and the role of vitamin E in preventing this cytotoxicity.

**Methods:**

Thirty adult male albino rats were randomly divided into 3 groups: Negative control received olive oil for 3 weeks; Study I received olive oil for 1 week, then daily oral administration of 300 mg/kg TiO_2_NPs for 2 weeks; and Study II received 100 mg/kg vitamin E diluted in 100 ml olive oil daily as a prophylactic from day 1 for 3 weeks. On day 8, with vitamin E, they received 300 mg/kg TiO_2_NPs for 2 weeks by oral gavage. All samples were examined via hematoxylin & eosin (H&E), histomorphometry of serous acinar surface areas, transmission electron microscopy (TEM), and blood analysis of malondialdehyde (MDA) and interleukin (IL-1β) levels.

**Results:**

Serum levels of both MDA and IL-1β were significantly greater in study I than in control and study II groups. Histologic examination revealed structural changes in serous acini and ducts of study I, with great preservation of the normal appearance of the acini and ducts in study II. Histomorphometry revealed a significant difference between control and study I, with no significant difference from that in study II. TEM revealed multiple ultrastructural changes in acinar cells and ducts of study I compared with those of study II, which maintained their normal features.

**Conclusions:**

Vitamin E plays crucial antioxidant and anti-inflammatory roles in counteracting the cytotoxic effects of TiO_2_NPs by alleviating their deleterious impact on salivary glands.

**Supplementary Information:**

The online version contains supplementary material available at 10.1186/s12903-025-06631-w.

## Background

With the rapid advancement of nanotechnology and its broader applications over the past decade, nanoscience has prosperously increased. Materials at the submicrometer scale (1–100 nm) are known as nanomaterials, and they are widely used in a variety of products. Titanium dioxide nanoparticles (TiO_2_NPs) are commonly used in medicine, dentistry, and the food industry. They have been employed as white pigments in various products, such as sunscreens, toothpastes, dental restoration materials, antibacterial agents, and drug delivery systems [1]. 

Titanium dioxide (TiO_2_) is frequently used in food, including as a food additive E171 in chewing gum, candies, and coffee creamers [2]. The amount of pigmentary TiO_2_ used annually is close to 4.6 million tons, and this figure has increased as consumption has increased. There are worries that exposure to this drug over an extended period could have negative consequences on the human body [3]. Due to the low dissolution rate of TiO_2_NPs, their cytotoxic effects are likely related to their size rather than the release of metallic ions [3]. According to the Centers for Disease Control and Prevention (CDC) and the National Institute for Occupational Safety and Health (NIOSH), there are two different occupational exposure limits for TiO_2_ particles on the basis of their size: fine TiO_2_NPs (> 100 nm) and ultrafine TiO_2_ (< 100 nm). NIOSH recommends exposure limits of fine and ultrafine TiO_2_ to be 2.4 mg/m3 and 0.3 mg/m3, respectively [2, 4]. 

The extensive ingestion of TiO_2_NPs promotes the expression of proinflammatory genes and has genotoxic effects. The formation of excessive reactive oxygen species (ROS) on the surface of TiO_2_NPs causes oxidative stress, thus leading to inflammation, cytotoxicity, genotoxicity, immunotoxicity, and neurotoxicity [3, 5]. TiO_2_NPs bind to the cell membrane and intracellular organelles via electrostatic interactions, causing cellular damage and lipid peroxidation of the cell membrane [6]. Moreover, the internalized TiO_2_NPs cause protein adsorption, thus blocking signaling pathways and binding to DNA structures, causing damage, rearrangement, and altered gene expression [3, 6]. 

Oxidizing and reducing agents interact with O_2_ to form on the reactive surface of NPs, leading to additional production of reactive oxygen species (ROS). Exciting TiO_2_NPs present in aqueous environments reduce O_2_ molecules, thus generating radical species such as hydroxyl radicals via the oxidation of H_2_O and superoxide radicals. Redundant accumulation of ROS initiates the action of cytokines and proinflammatory mediators, which control the transcription of inflammatory genes such as interleukin-1 beta (IL-1β), IL-8, and tumor necrosis factor-alpha (TNF-α) [7]. 

The imbalance between ROS and antioxidants produced by oxidative stress leads to mitochondrial dysfunction, ultimately affecting cellular vitality [8, 9]. The ability of endogenous and dietary antioxidants to function as preventative measures against damage caused by various types of NPs by reducing the level of ROS makes them the first line of defense [10]. 

Several types of nonenzymatic antioxidants, such as vitamins A, C, D, and E, are used to treat oxidative stress [11]. As a peroxyl radical scavenger, vitamin E is a dietary antioxidant that has been shown to reduce oxidative stress by slowing the oxidative cascade, inhibiting the formation of new free radicals and neutralizing existing ROS, thus protecting organs under stress [12, 13]. 

Owing to the lipophilic nature of vitamin E, it prevents the peroxidation of unsaturated fatty acids in the cell membrane [7]. Vitamin E plays a role in the suppression of inflammatory and oxidative stress by downregulating TNF-α, IL-1, and IL-6 [7]. The oral ingestion of TiO2NPs through diet or environmental exposure poses health risks, and research on its potential risks to oral health has been limited.

TiO_2_NPs can pass through biological barriers such as the epithelium, mucosa, and endothelium. The gastrointestinal tract is the main route of passage for TiO_2_NPs. Research has shown that TiO_2_NPs enter the bloodstream and accumulate in and affect different organs, such as the kidney, liver, and salivary glands [14]. 

The literature has focused on the effects of different types of nanoparticles, such as silver [15–17], zinc oxide [18], and copper oxide [19], on salivary glands. In addition, the lingual mucosa may experience cytotoxic effects from a low dose and prolonged exposure period of TiO_2_NPs [20]. However, studies assessing the antioxidant properties of vitamins such as vitamin E and their ability to reduce or even reverse the consequences of oxidative stress induced by TiO_2_NPs on the submandibular salivary glands in experimental animal models are limited.

Therefore, the objective of the current study was to examine the impact of TiO_2_NPs on submandibular salivary glands and the potential role of vitamin E supplementation in preventing any cytotoxicity caused by TiO_2_NPs to further understand their effects in the oral cavity.

## Methods

### Study design (in vivo study)

The number of animals in this study was estimated on the basis of calculations of the sample size made in the Department of Biomedical Informatics and Medical Statistics, Medical Research Institute, Alexandria University. A power of 80% (β = 0.20) was used to detect a standardized effect size in the surface area of the salivary gland acini (primary outcome), and a level of significance of 5% (α error accepted = 0.05) was used. The minimum required sample size was 8 rats per group (number of groups = 3). (total sample size = 24 rats), as guided by a prior study conducted by Afshari-Kaveh et al. [21, 22].

### Settings

For the Department of Biomedical Informatics and Medical Statistics, the sample size was calculated via GPower version 3.1.9.2 [23].

**Study subjects**:

Thirty adult male albino rats weighing 200–250 g were obtained from the experimental animal house of the Medical Research Institute, Alexandria University. The animals were randomly assigned via computer-generated random numbers to one of 3 groups, with 10 rats per group (total sample size = 30 rats).

Before the study began, the rats were allowed to adapt for 10 days, with free access to water and a standard, pelleted diet. At the beginning of the study, the animals were weighed to calculate the doses of TiO_2_NPs and vitamin E. Additionally, they were weighed weekly during the study to ensure their health. The animals were housed in specially designed wire mesh bottom cages, with 3 rats per cage, at room temperature and maintained under a light‒dark cycle (L: D 12:12 with lights on at 07:00 h).

- **In the control group**, 10 rats received olive oil by oral gavage daily for 3 weeks (negative control).
- **In the study I group**, 10 rats received olive oil for 1 week and then 300 mg/kg TiO_2_NPs by oral gavage daily for 2 weeks [24–26].
- **In the study II group**, 10 rats received 100 mg/kg vitamin E (α-tocopherol) [15]diluted in 100 ml of olive oil daily as a prophylactic from day 1 for 3 weeks. On day 8 with vitamin E, they received 300 mg/kg TiO_2_NPs for 2 weeks by oral gavage, which means that vitamin E administration began one week before TiO2NP exposure [21, 27].

### Materials

1. Titanium dioxide NPs^1^ in powder form (anatase phase) were obtained from Nanogate Company.
2. Vitamin E^2^ in the form of capsules was obtained from Khalil Pharmacy.

### Titanium dioxide NP Preparation and characterization

**Fig. 1:**
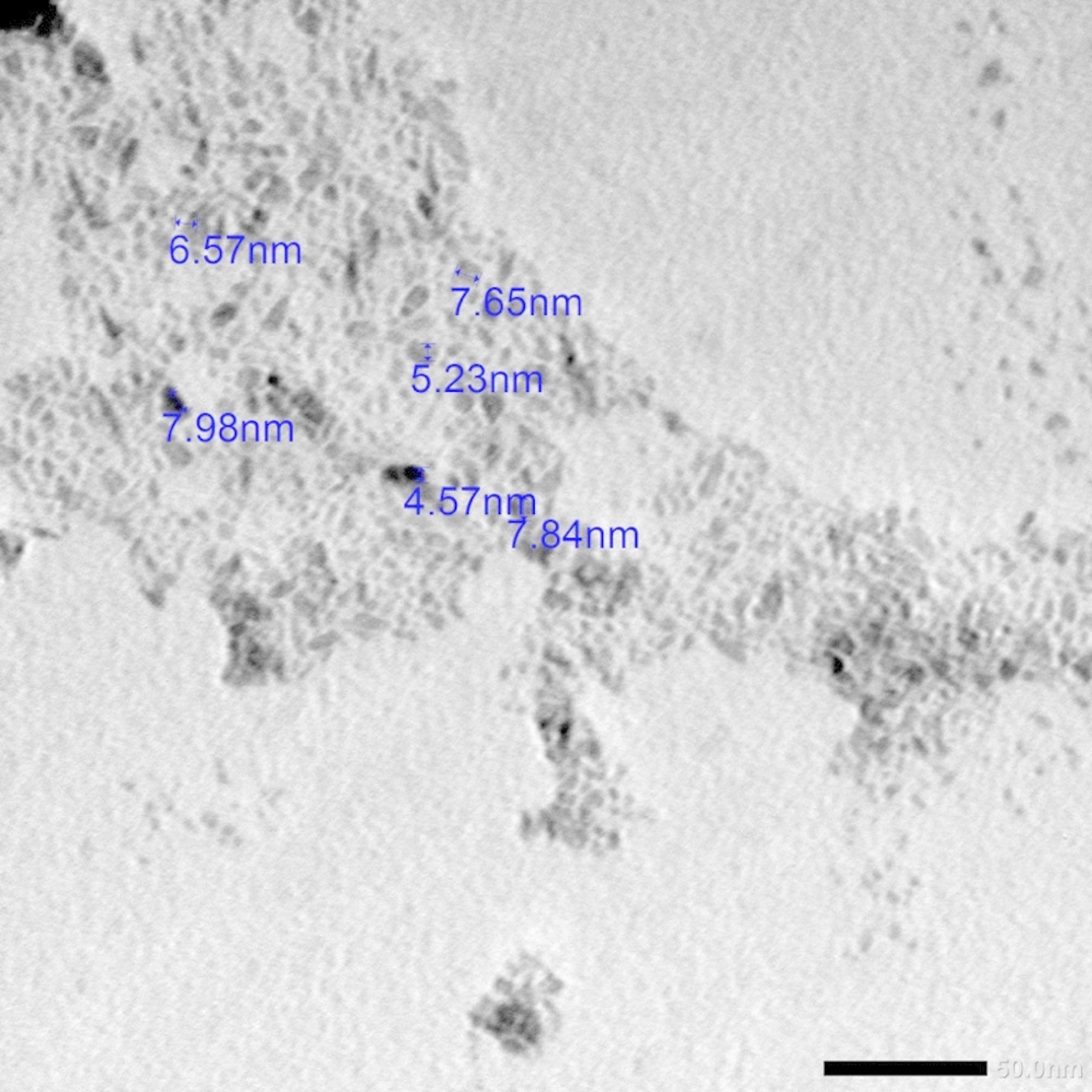
Transmission electron micrograph (TEM) showing TiO_2_NPs less than 100 nm in size (TEM ×100,000. Scale bar 50 nm)

Titanium dioxide NPs are available in powder form. They were suspended in distilled water and administered orally via oral gavage to study groups I and II for 2 weeks. To study the size and morphology of the particles, the TiO_2_NPs were characterized. The suspension of the nanoparticles was drop-cast onto a carbon-coated copper grid, which was then air-dried at room temperature and visualized via a JEOL transmission electron microscope (Japan). The examination was performed in the Faculty of Science, Alexandria University. Particle size measurements revealed that the TiO_2_NPs had sizes between 5 and 8 nm (Fig. 1).

### Vitamins E administration

Animals in the study II group received vitamin E (-tocopherol) 100 mg/kg daily as a prophylactic starting on day 1 for three weeks. They received 300 mg/kg TiO_2_NPs by oral gavage on day 8 for two weeks [21, 27], and their administration volume was set at 10 ml/kg bw to prevent aggregation [24]. 

Vitamin E was obtained from Pharco Pharmaceuticals, Cairo, Egypt, as 1 gm capsules were dissolved in 100 ml of olive oil [28]. All the supplements were prepared just before administration [28]. Both supplements (TiO_2_NPs and vitamin E) were prepared just before administration.

The no observed adverse effect level (NOAEL) of vitamin E was 125 mg/kg/day in a 13-week oral toxicity trial in rats, according to the European Food Safety Authority [27, 29]. Therefore, a lower dose (100 mg/kg) was utilized in this study.

### Animal euthanasia

At the end of the experiment (3 weeks), the rats were euthanized. The animals were generally anesthetized via isoflurane to guarantee that all the animals were painless and euthanized. Well-trained veterinary staff performed cervical dislocation in the animal facility located in the Faculty of Medicine, Alexandria University. The method used abided by the American Veterinary Medical Association (AVMA) Guidelines for the Euthanasia of Animals (2020 Edition) [30]. 

### Assessment

#### Blood analysis

Blood samples were collected from the abdominal aorta of the euthanized rats into plain vacuum tubes and allowed to clot at room temperature for the detection of MDA and IL-1β in the blood serum. Blood samples were centrifuged for 10 min at 6000 rpm.

### Measurement of MDA

The MDA colorimetric assay was carried out as reported by Ohkawa, H. Ohishi W, and Yagi K., where MDA reacts with thiobarbituric acid in acidic medium at 95 °C to form a thiobarbituric acid-reactive pink color product. The absorbance of this product was measured at 534 nm and was positively correlated with the MDA concentration [31]. 

### Interleukin (Il)-1-BETA ELISA

The IL-1 β concentration in the serum was determined via a Rat Aviva Systems Biology Kit (Aviva Systems Biology, Corp., San Diego, USA) according to the manufacturer’s instructions [32]. 

#### Histological procedures

Salivary gland tissue from the right submandibular area was processed for light microscopic examination. The samples were fixed in 10% neutral buffered formalin (Sigma‒Aldrich Co.) for 48 h. The samples were dehydrated in increasing concentrations of ethanol, infiltrated, and then embedded in paraffin wax. Sagittal 4 μm thick sections were stained with hematoxylin and eosin (H&E) (Sigma‒Aldrich Co.) for histological evaluation and histomorphometric analysis.

#### Histomorphometric analysis: [33]

The surface area (SA) of the serous acini was measured in millimeters squared (mm^2^). For each animal, 2 serial sections were obtained, and 5 serous acini were randomly selected for each measurement. The selected acini were cross-sectional and had a circular circumference for a standardized measurement of the surface area. The measurements were estimated in photos captured at x400 magnification with the aid of an image analysis system (ImageJ).

#### Transmission electron microscope

Tissues from the left submandibular salivary glands were cut into small pieces and fixed in 2.5% glutaraldehyde for 24 h. The tissue segments were washed thoroughly in phosphate buffer (0.1 M, pH 7.4). Postfixation of the tissues was performed in freshly prepared buffered 1% osmium tetroxide in phosphate buffer (Sigma‒Aldrich Co.) for 1.5 h at 4 °C. After postfixation, the tissues were washed in phosphate buffer (three changes each for 10 min) to remove the excess osmium tetroxide [34]. The extraction of water was accomplished by washing the sample in ascending grades of ethyl alcohol. The samples were embedded in Beem capsules. Ultrathin sections of approximately 60 nm thickness were cut using an LKB microtome. The chosen sections were then mounted on copper grids. The sections were examined, and photographs were captured via a JEOL transmission electron microscope (Japan). The examination was performed in the Faculty of Science, Alexandria University.

### Statistical analysis

Data were collected and analyzed via the SPSS (Statistical Package for Social Science) program for statistical analysis (ver 21).

The Shapiro–Wilk test of normality was used to assess the distribution of the quantitative variables. Most of the continuous variables were not normally distributed, so nonparametric statistics were used. The data are presented as the minimum, maximum, median, 95% CI of the median, and 25th to 75th percentiles (interquartile range (IQR)) [35]. Post hoc pairwise comparisons for which the Kruskal‒Wallis test or Friedman test was significant were performed via the Dunn‒Sidak test for multiple comparisons. The P value was adjusted via the Bonferroni correction method. The beta error reached 20%, with a power of 80%. The alpha level was set to 5%, with a significance level of 95%. Statistical significance was tested at p values < 0.05.

## Results

### Blood levels

**Fig. 2:**
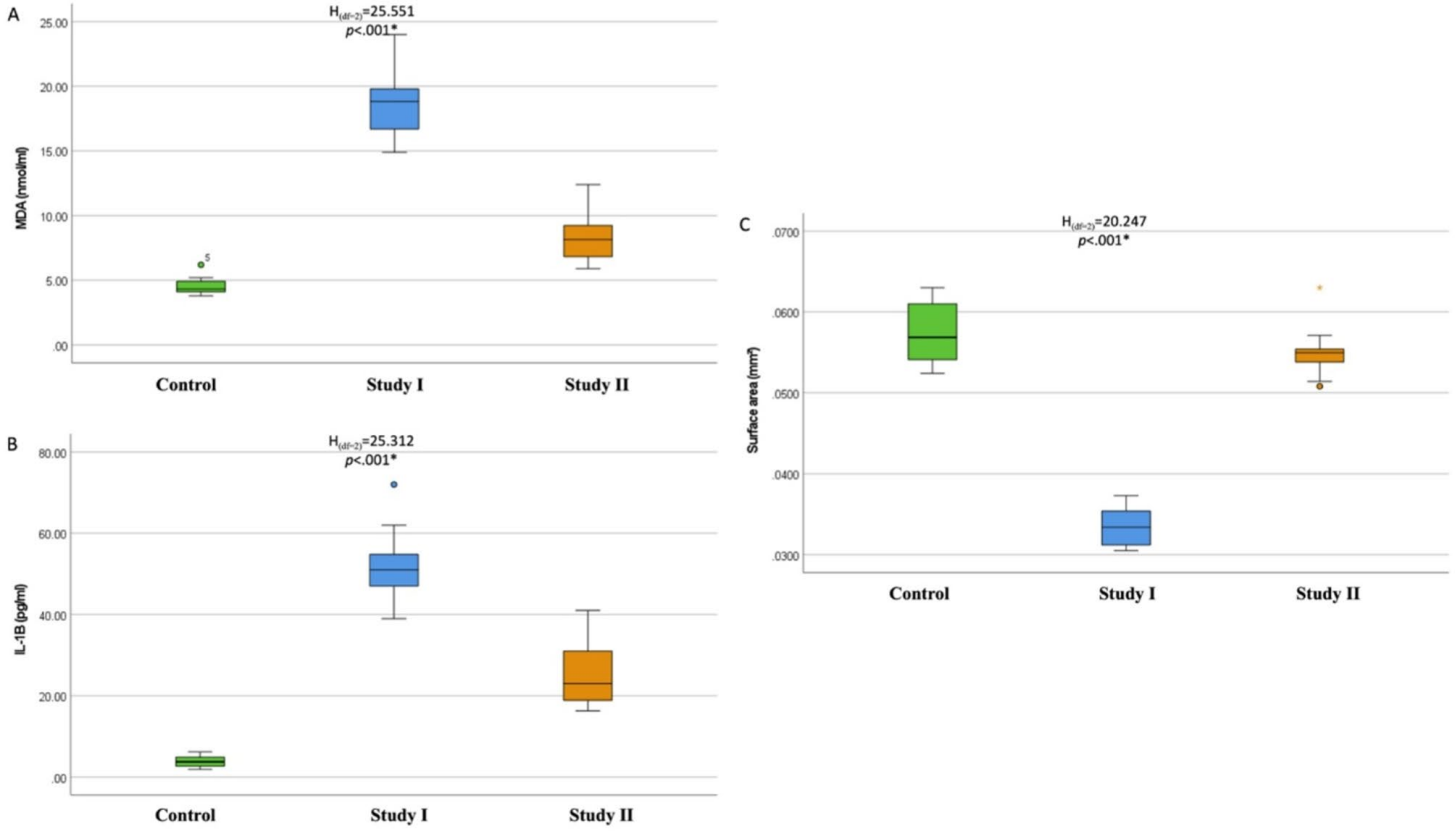
Box and whisker graph. The thick line in the middle of the box represents the median, the box represents the interquartile range (from the 25th to 75th percentiles), and the whiskers represent the minimum and maximum values. 2A: MDA (nmol/ml) serum levels, 2B: interleukin 1-beta (IL-1β, pg/ml) serum levels, and 2 C: mean surface area (SA) of the serous acini (mm^2^)

The present study explored the effects of TiO2NP exposure with and without the prophylactic effects of vitamin E on the serum levels of the lipid peroxidation product malondialdehyde (MDA, mmol/ml) and the proinflammatory cytokine interleukin 1-beta (IL-1β, pg/ml). The serum levels of MDA were significantly greater in Study I than in the control group, with a mean ± SD of 18.56 ± 2.99 *(p* <.001). Study II showed significantly lower levels than did Study I, with a mean ± SD of 8.50 ± 2.02 *(p* <.05). There was a significant difference between the control group and study II, where the control group had a mean ± SD of 4.55 ± 0.77 *(p* <.05). (Fig. 2A)

IL-1 β serum levels were significantly greater in Study I, with a mean ± SD of 51.78 ± 9.88, than in the control group *(p* <.001). Study II showed significantly lower levels than did Study I, with a mean ± SD of 25.32 ± 8.37 *(p* <.05). There was a significant difference between the control group and Study II, where the control group had a mean ± SD of 3.98 ± 1.39 *(p* <.05). (Fig. 2B)

### Light microscopic examination

**Fig. 3:**
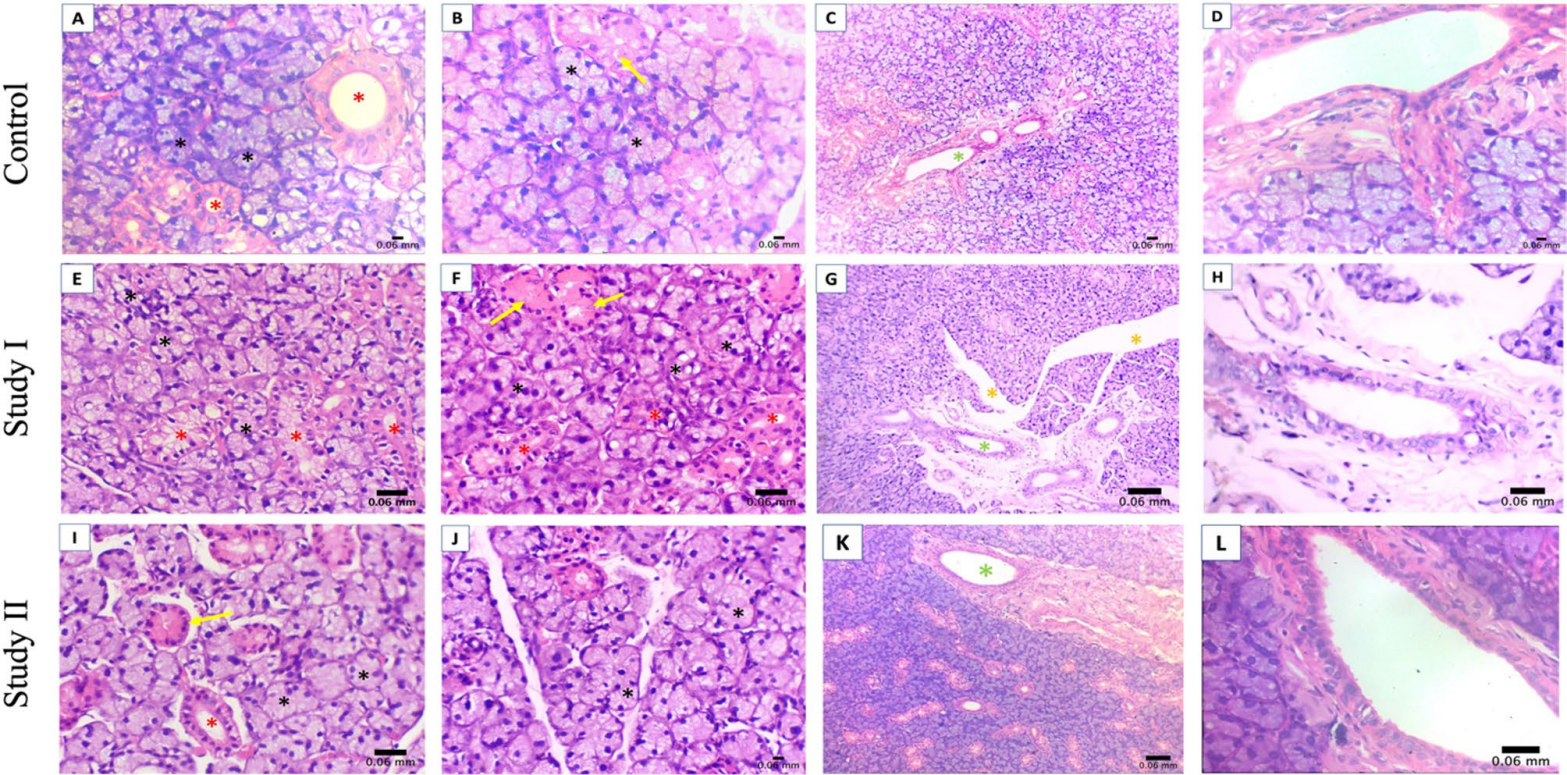
Light micrographs (LM) of a rat submandibular gland, H&E stain, control [3 A, 3B, 3 C, 3D], (3 A, 3B) serous acini (black asterisk), striated ducts (red asterisk), and granular convoluted ducts (yellow arrow) ×400. (3 C) Excretory duct (green asterisk) ×100. (3D) Higher magnification of the excretory duct ×400. Study I [3E, 3 F, 3G, 3 H], (3E) Nuclear pleomorphism and abnormal appearance of serous acini (black asterisk) striated ducts (red asterisk) x400. (3 F) Abnormally granular convoluted ducts (yellow arrow). Nuclear pleomorphism of serous acini (black asterisk) and striated ducts (red asterisk) x400. (3G) Degeneration of fibrous tissue (green asterisk) ×100. Note the large interacinar spaces (orange asterisk). (3 H) Higher magnification image of the excretory duct ×400. Study II (3I, 3 J, 3 K, 3 L). (3I, 3 J) Serous acini (black acini), striated (red asterisk), and granular convoluted ducts (yellow arrow) x400. (3 K) Excretory duct (green asterisk) ×100. (3 L) Higher magnification of the excretory duct x400

The control group presented normal serous acini that were spherical and consisted of pyramidal cells. Both striated and granular convoluted ducts had a normal appearance (Fig. 3A and B). In addition, the excretory ducts exhibited a normal appearance and were surrounded by dense fibrous interacinar tissue (Fig. 3C and D). Study I revealed nuclear pleomorphism and abnormal histological features of both serous acini and striated ducts (Fig. 3E). The convoluted granular ducts appeared ill-defined (Fig. 3F). The excretory ducts exhibited degeneration of the surrounding fibrous tissue, and large interacinar spaces were observed (Fig. 3G and H). In the study II group, the normal appearance of serous acini, striated, and granular convoluted ducts was preserved to a great extent (Fig. 3I and J). Moreover, the dense fibrous interacinar tissue of the excretory ducts was preserved. (Figure 3K and L)

### Histomorphometric analysis

The mean surface area (SA) of the serous acini (mm^2^) was significantly smaller in Study I, with a mean ± SD of 0.0334 ± 0.0023, whereas Study II presented significantly greater SAs than did Study I, with a mean ± SD of 0.0550 ± 0.0034. There was no significant difference between the control group and study II, where the control group had a mean ± SD of 0.0574 ± 0.0037. (Fig. 2C)

### Transmission electron microscopy (TEM)

**Fig. 4:**
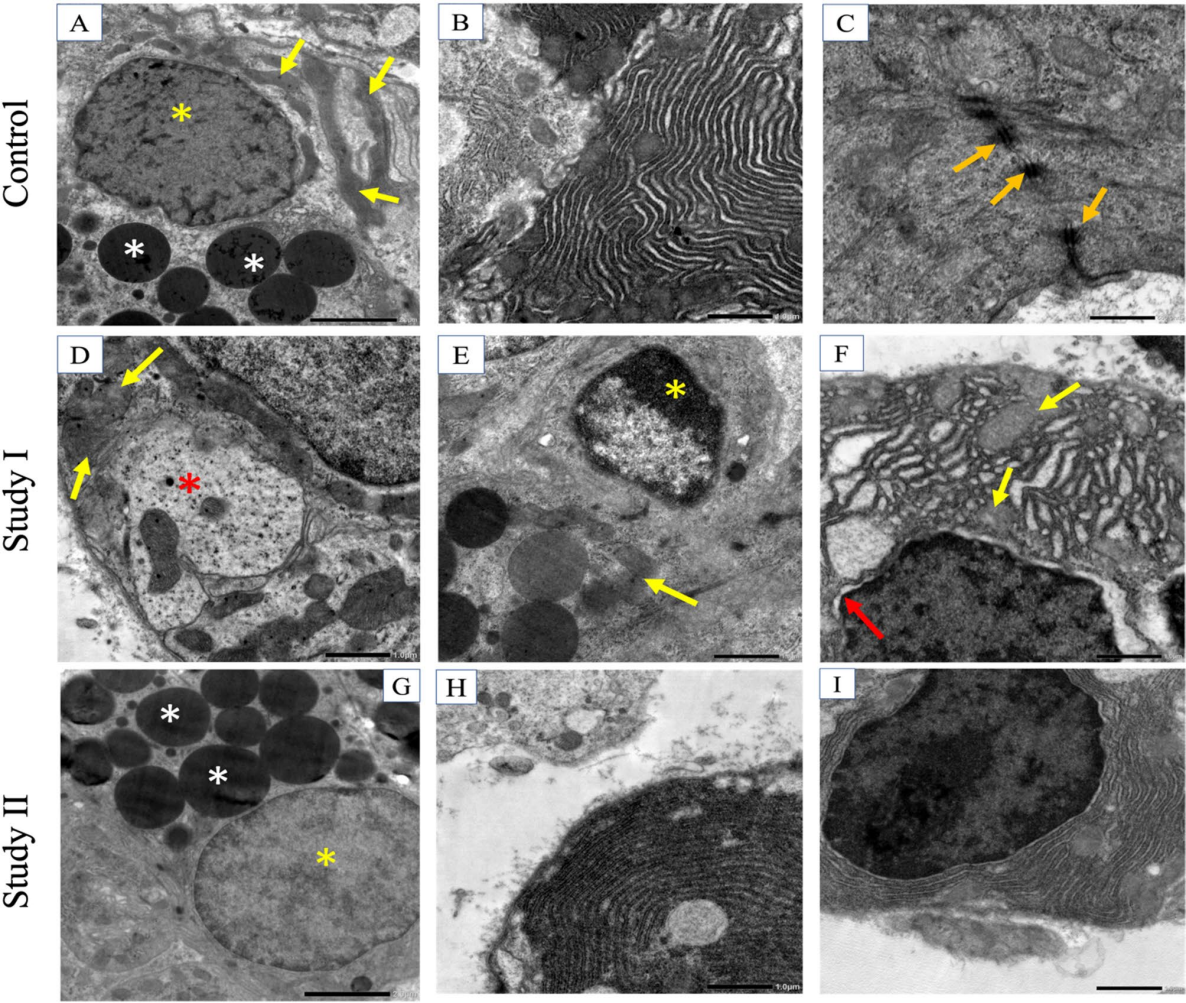
TEM image showing the salivary acini of the control (4 A, 4000×) active nucleus of serous acini (yellow asterisk) and uniform secretory globules (white asterisk). mitochondria present perinuclearly (yellow arrows). (4B, x6000) rER. (4 C, ×12000) Cellular junctions (orange arrows). Study I (4D, ×6000) serous acinar cells revealing cytoplasmic degeneration (red asterisk). Loss of mitochondrial cristae (yellow arrows). (4E, x6000) Chromatin condensation (yellow asterisk). Loss of mitochondrial striations (yellow arrow). (4 F, ×6000) Abnormal appearance of the rER surrounding the nucleus of the acinar cell along with loss of the striations of mitochondria (yellow arrows) and blebbing of the nuclear membrane (red arrow). Study II (4G, x4000) revealed the active nucleus of the acinar cell (yellow asterisk) and secretory granules (white asterisk). (4 H, 4I, ×6000) Normal appearance of the rER

**Fig. 5:**
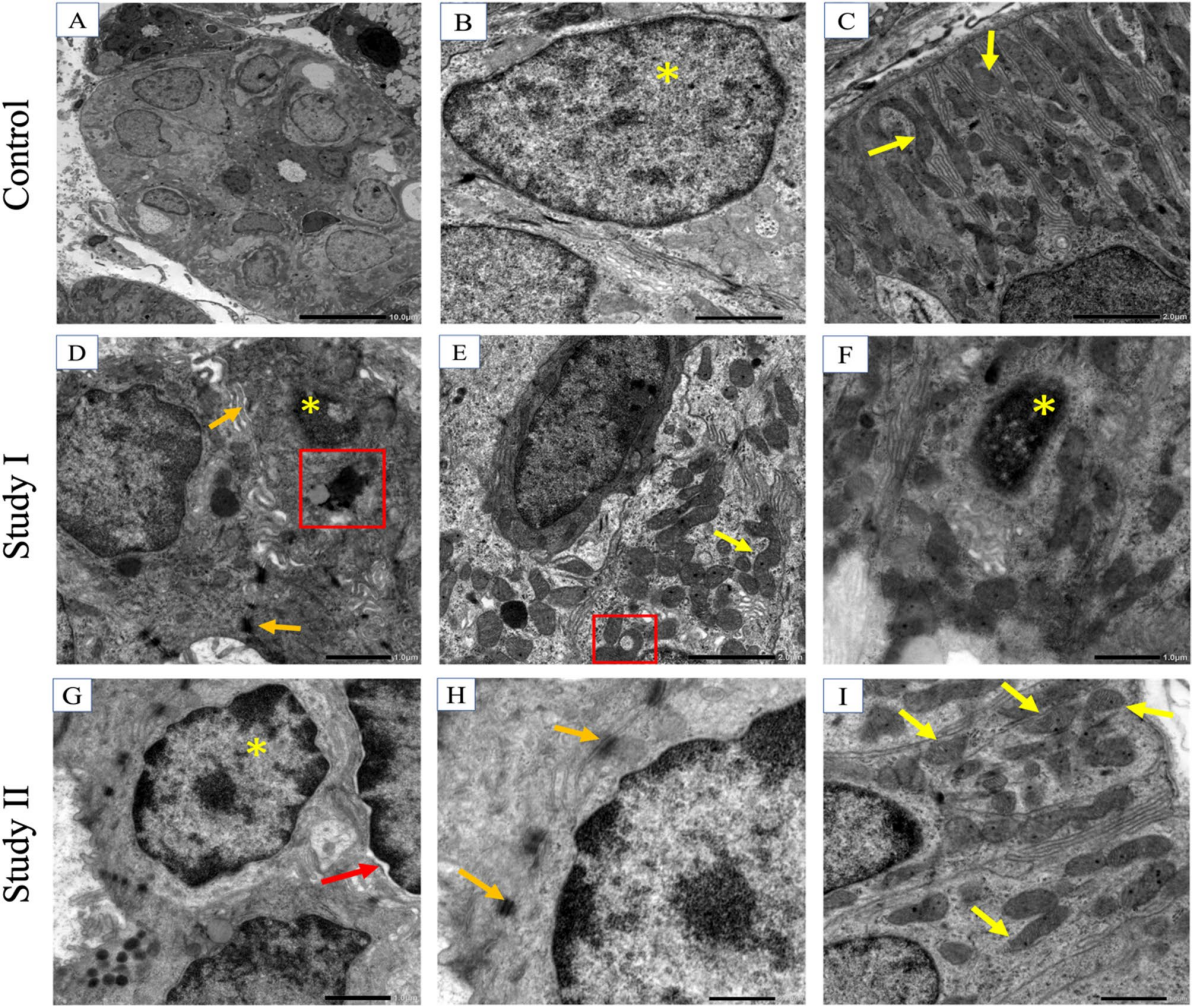
TEM image showing salivary ducts, control, (5 A, x800) intercalated duct, (5B, x4000) active nucleus (yellow asterisk) of the intercalated duct, (5 C, x4000) striated duct showing distinct basal foldings with regularly organized mitochondria (yellow arrows). Study I, (5D, ×6000) Intercalated duct with pyknotic apoptotic nucleus (yellow asterisk), tertiary lysosome (square area), and abnormal intercellular attachment (orange arrows). (5E, x4000) Striated duct showing loss of basal striations, disorganized apoptotic mitochondria (square area), and irregularly shaped fused mitochondria (yellow arrow). (5 F, ×6000) Pyknotic nucleus (asterisk) of the striated duct. Study II: (5G, x6000) Active nuclei of the intercalated duct (yellow asterisk), separation between the nucleus and cytoplasm (red arrow). (5 H, ×12000) Higher magnification of the previous image showing intact cellular membranes and desmosomes (orange arrows). (5I, x6000) Striated duct with evident basal striations. Well-defined cristae of the mitochondria (yellow arrows)

The acinar cells in the control group presented active euchromatic nuclei with surrounding cytoplasm containing uniform secretory granules (Fig. 4A). The perinuclear region contained abundant mitochondria and a regularly organized rough endoplasmic reticulum (rER) (Fig. 4B). Moreover, the cellular junctions between acinar cells exhibited a regular organization (Fig. 4C). The intercalated ducts presented normal features and an active nucleus (Fig. 5A and B). In addition, the striated ducts exhibited distinct basal folding with regularly organized mitochondria (Fig. 5C).

In the study I group, serous acinar cells exhibited several regions of cytoplasmic degeneration (Fig. 4D). In addition, abnormally organized mitochondria with loss of characteristic striations were frequently observed (Fig. 4D, E and F), as was the abnormal appearance of the rER surrounding the nucleus of acinar cells (Fig. 4F). Nuclear changes were also detected. Some cell nuclei revealed apoptotic degeneration along with an increase in chromatin condensation at the nuclear edges (Fig. 4E). Moreover, nuclear blebbing was detected where the outer layer of the nuclear membrane extended toward the cytoplasm, causing perinuclear separation from the cytoplasm (Fig. 4F).

The cells of the intercalated duct of the study I group were irregular in their outline. They also revealed pyknotic apoptotic nuclei (Fig. 5D). The intercellular attachments were negatively affected and appeared disrupted (Fig. 5D). Tertiary lysosomes were also detected (Fig. 5D and E). Moreover, the striated duct showed a loss of basal striations and disorganized, irregularly shaped fused mitochondria (Fig. 5E and F). Some of the mitochondria exhibited signs of apoptosis (Fig. 5E).

The acinar cells of the study II group presented active euchromatic nuclei with regular outlines (Fig. 4G). The perinuclear region exhibited a regularly organized rER (Fig. 4H and I). The intercalated ducts preserved their normal features and an active nucleus. Most of the nuclei were not distinguishable from the cytoplasm, with one nucleus revealing a lesser extent of perinuclear spacing in comparison to that in the Study I group (Fig. 5G). The intercellular attachments preserved their regular organization (Fig. 5H). In addition, the striated ducts preserved their distinct basal folding with regularly organized mitochondria and well-defined cristae (Fig. 5I).

## Discussion

The use of nanoparticles (NPs) has extensive virulence effects on various tissues. In the present study, we focused on the impact of TiO2NPs on submandibular salivary glands and the role of vitamin E in protecting against the cytotoxicity of TiO_2_NPs.

Previous investigations revealed that rodents require 50 to 500 mg/kg body weight TiO_2_NPs to cause acute toxicity [36, 37]. Although some research has indicated that toxicity occurs at relatively low concentrations, toxicity in such quantities cannot be generalized across a wide range of parameters. According to previous research, the biochemical and histological characteristics of the spleen [21] and liver [7, 38] of Wistar rats can change when they are exposed to 300 mg/kg TiO_2_NPs. They concluded that to ensure the production of definite acute toxicity in rats, 300 mg/kg TiO_2_NPs were utilized.

TiO_2_ powder with an anatase phase, a tetragonal crystalline form with a mean particle size of less than 10 nm, was used in the current investigation. Weir et al. reported genotoxicity caused by TiO_2_NPs with a diameter of less than 25 nm in the anatase crystalline phase [39]. Compared with other forms, such as rutile, the anatase form of titanium dioxide nanoparticles is more cytotoxic and phototoxic [6, 40, 41]. 

Antioxidants, also known as free radical scavengers, contain unpaired electrons. This allows them to reduce or prevent damage caused by free radicals. Vitamin C is a potent water-soluble antioxidant that is acquired exogenously. Vitamin E (α-tocopherol) is a membrane-bound antioxidant. Since it is a lipid-soluble vitamin, it prevents lipid peroxidation of the cell membrane, enhances membrane stability, and maintains its fluidity [11]. 

Shamel et al. examined the efficacy of vitamin C in preventing submandibular gland toxicity caused by copper oxide nanoparticles (CuO-NPs). CuO-NPs significantly damaged the submandibular salivary glands of albino rats, with increased levels of Ki-67 and caspase-3 compared with those in the control group. These damaging effects were successfully diminished by vitamin C supplementation. A decrease in the levels of Ki-67 and caspase-3 in the vitamin C/CuO-NP group, along with an improvement in salivary tissue histological features, indicates its ability to counteract ROS production [19]. 

Bakr et al. considered vitamin D an antioxidant due to its role in preventing the development of oral mucositis [42]. It inhibits the expression of nicotinamide adenine dinucleotide phosphate (NADPH) oxidase and increases the expression of several antioxidant molecules, including glutathione, glutathione peroxidase (GPx), and superoxide dismutase (SOD). Additionally, it increases the production of anti-inflammatory cytokines such as IL-10 and decreases the expression and production of various proinflammatory cytokines such as TNF-α, IL-1β, IL-6, and IL-8 [21]. Therefore, vitamin supplementation may be implemented to counteract the destructive effects induced by oxidative stress.

The serum levels of MDA in the current study were substantially greater in Study I than in the control group, indicating the impact of TiO_2_NPs on lipid metabolism and peroxidation. On the other hand, Study II showed considerably lower levels than did Study I, indicating the prophylactic role of vitamin E. In 2020, a controlled study was conducted in which rats presented increased MDA serum levels and decreased SOD activity after TiO_2_NP exposure in comparison with those in the control group [2]. In another study, rats were orally administered 300 mg/kg TiO_2_NPs for 14 days. Compared with the control, the administration of TiO_2_NPs significantly increased the MDA levels in the liver [38]. Shirdare et al. reported increased serum MDA levels in rats given a dose of 300 mg/kg TiO_2_NPs dissolved in olive oil [43]. All the previously mentioned studies revealed an increase in MDA serum levels, as observed in the present study, after the administration of TiO_2_NPs.

Moradi et al. [7]. Vitamin E (100 IU/kg) was administered to the rats 7 days before the administration of TiO_2_NPs on day 8. The rats were treated with 300 mg/kg TiO_2_NPs for two weeks via oral gavage, followed by continual administration of vitamin E for another 2 weeks. Compared with those in the control group, the serum MDA levels in the TiO_2_NPs group were significantly greater. On the other hand, the vitamin E-supplemented group presented a significant decrease in MDA levels. Moreover, an increase in TNF-α levels was observed in the TiO_2_NPs group, unlike in the vitamin group, where TNF-α levels were significantly decreased [7]. These findings were in accordance with those of the current study.

In the present study, the levels of IL-1 β were significantly greater in Study I, whereas Study II presented significantly lower levels than did Study I. In contrast to our findings, Elgrabli et al. reported that TiO_2_ exposure did not affect the serum IL-1β or IL-6 levels [32]. This can be attributed to the use of very low intravenous doses at concentrations ranging from 7.7 to 9.4 mg/kg. On the other hand, the findings of Pettersson et al. were in accordance with the current results [44]. The secretion of IL-1β increased in a dose-dependent manner in cells exposed to the titanium ion mixture. The bioactive protein aggregates formed by internalized titanium NPs with calcium and phosphorus trigger an immune response via the secretion of various inflammatory mediators, such as IL-1β, IL-6, and tumor necrosis factor-alpha (TNF-α). Moreover, Ramenzoni et al. revealed that macrophage cultures presented elevated expression levels of the inflammatory cytokine TNF-α and of IL-1β 12, 24, and 48 h after exposure to TiO_2_NPs [45]. Baranowska-Wójcik et al. reported that TiO_2_NPs increased the secretion of both IL-1β and TNF-α in Caco-2 colon cancer cell culture [46]. 

Several studies have assessed the levels of enzymatic antioxidants after the administration of NPs and the protective role of vitamins A and E. Afshari-Kaveh et al. reported that the gene expression of SOD in the TiO2NP-consuming group was substantially lower than that in the control group (*p* <.001). In contrast, the rats treated with vitamins A and E separately or in combination presented a significant increase in the gene expression of SOD compared with that in the TiO2NP group (*p* <.05). However, the alleviative effects of vitamins are insufficient for restoring normal levels of gene expression [21]. Moreover, according to Bayat et al., the livers of ZnO-treated rats presented substantial decreases in antioxidant enzyme activity and the gene expression of SOD, glutathione peroxidase (GPx), and catalase compared with those of the control group (*p* <.05). On the other hand, vitamin E supplementation may considerably alter the previously described alterations and lessen the oxidative stress caused by ZnO NPs [47]. 

In the present study, the I group revealed an abnormal histological appearance of both serous acini and ducts. Meanwhile. In the study II group, the normal appearance of the acini and ducts was preserved. The mean surface area (SA) of the serous acini was found to be significantly smaller in Study I. At the same time, Study II was considerably larger than that of Study I.

In 2023, a study in which rats were injected with TiO_2_NPs was conducted. Abdel Aal et al. [16] found that NPs increased fibrosis and acinar cell apoptosis in the parotid salivary glands. The negative effects of TiO_2_NPs were less severe than those of AgNPs. However, discontinuation of exposure to both types of NPs alleviated the damage to the histological findings. Another study was conducted by Abdel Aal et al. to assess the effects of TiO_2_NPs on the pancreas of adult male rats. The serous acini of the control group were closely packed with well-defined boundaries. The rats treated with 150 mg/kg TiO_2_NPs for 14 days presented distorted acini with irregular borders [1]. 

To the best of our knowledge, no studies have evaluated the ability of vitamin E to attenuate the negative histological effects of TiO_2_NPs on salivary glands. However, Zaki et al. studied the effects of vitamin E on AgNPs and groups. The vitamin E-treated group presented an almost normal ultrastructural architecture of both the acini and ducts, with almost no cytoplasmic vacuolization. In addition, the striated ducts regained their basal striations [15]. 

Ultrastructural examination via TEM revealed cytoplasmic degeneration of serous acinar and ductal cells and mitochondrial swelling in study I. Moreover, nuclear changes, as well as apoptotic degeneration, were detected. Furthermore, the intercellular attachments appeared disrupted. These observations are in accordance with Abdel Aal et al. [16], who reported that NPs damage intercellular tight junctions by upregulating the expression of inflammatory cytokines, generating oxidative stress, and disrupting the Occludin protein. Another in vitro study reported that tight junctions in the intestinal epithelium were negatively impacted by TiO_2_NPs after a 4 h incubation time [48]. 

Mitochondrial dysfunction and cellular apoptotic signs can be related to the excessive production of ROS, which is the hallmark of oxidative stress damage in many pathologies [49]. This finding correlates with the elevated MDA and IL-1β reported in Study I. Moreover, the vacuolations observed in the rER, which is an essential contributor to the synthesis and folding of secreted and membrane-bound proteins, indicate a cellular imbalance. An imbalance between the protein-folding ability of a protein and its functional demand can induce increased ER stress, resulting in cell apoptosis [49]. 

For the in vivo experiments, the mice were orally exposed to 200 or 500 mg/kg TiO_2_NPs for 90 days. Both in vitro and in vivo flow cytometric analysis revealed that cell viability decreased with increasing doses of TiO2NPs [50]. In another published paper, control murine macrophages presented normal structures of the cytoplasm, cellular organelles, and nucleus according to TEM. Moreover, the cells exposed to 400 mg/mL TiO_2_NPs for 24 h presented autophagocytic vacuoles [51]. Moreover, prolonged exposure to TiO_2_NPs induces the accumulation of autophagosomes and lysosomal impairment. This finding coincides with the detection of tertiary lysosomes in the study I group exposed to TiO_2_NPs in the present study [52]. 

On the other hand, the acinar and ductal cells of study II preserved their normal structural appearance, with active euchromatic nuclei and regular outlines. These results are in accordance with the findings of Zaki et al. [15]. TEM examination of submandibular salivary glands treated with vitamin E revealed enhancement of the acini and ducts, with results comparable to those of the control to a great extent. However, acini and ducts treated with AgNPs presented only signs of degeneration, abnormal cell organelles and various cytoplasmic vacuolations.

TiO_2_NPs are well known for their antimicrobial effects through the generation of reactive oxygen species (ROS) and thus disruption of bacterial cell walls. Moreover, the smaller the size of the nanoparticles is, the greater their antibacterial ability. However, this seems to be a paradoxical property. The small size of NPs facilitates their entry through the bloodstream into organs, cells, and even nerves via the digestive tract [49]. Their toxicity is inversely proportional to the size of the NPs [53]. 

Elevated levels of ROS and excessive production of free radicals cause adverse effects such as lipid and protein peroxidation, tissue damage, and mitochondrial dysfunction, ultimately leading to cytotoxicity and apoptosis [2]. 

Several studies have confirmed the cytotoxicity of most nanomaterials and their close association with inflammatory processes and the production of ROS [9, 16, 18, 54, 55]. TiO_2_NPs can also accumulate in macrophages, lung epithelial cells, and dermal fibroblasts [53]. 

Lipids are a fundamental part of the cellular structure and are involved in essential functions such as cellular homeostasis, along with cell‒cell communication [2, 7]. Another explanation for the essential role of vitamin E is that it elevates the lipid content of cell membranes, thus providing cellular stability and stabilizing cell membranes.

## Conclusions

Vitamin E can play a crucial antioxidant and anti-inflammatory role in attenuating the cytotoxicity induced by TiO_2_NPs and repairing cell membranes. TiO_2_NPs lead to an increase in MDA (a marker of lipoperoxidation) and elevated levels of IL-1β (an inflammatory cytokine). Prophylactic supplementation with vitamin E resulted in considerably lower levels of both MDA and IL-1β. Moreover, TiO_2_NPs negatively affected the histological architecture of both salivary serous acini and ducts. In addition, transmission electron microscopy (TEM) revealed that the nucleus, along with cellular changes, affected mainly the mitochondria and reR. In contrast, vitamin E succeeded in preserving the histological and ultrastructural features of both the salivary acini and ducts.

### Study limitations

Prolonged exposure to different doses of TiO_2_NPs should be examined to further understand the extent of their deleterious effects. Moreover, the impact of other antioxidants, such as vitamin D or C, can be assessed. Furthermore, the effects of the combination of vitamins C and E can be studied since they intensify the antioxidant properties of one another. In the present study, TiO2NPs led to an increase in MDA (a marker of lipoperoxidation) and elevated levels of IL-1β (an inflammatory cytokine). However, we recommend assessing the antioxidant response by measuring the levels of several enzymatic markers, such as SOD, GPx, and catalase. Further studies are recommended to examine mitochondrial dysfunction and ER stress as signs of oxidative stress induced by TiO_2_NPs and the role of vitamins in preventing further cellular damage and apoptosis.

## Electronic supplementary material

Below is the link to the electronic supplementary material.

Supplementary Material 1

Supplementary Material 2

## Data Availability

No datasets were generated or analysed during the current study.

## Abbreviations

CDC: Centers for Disease Control and Prevention
CuO-NPs: Copper oxide nanoparticles
GPx: Glutathione peroxidase
H&E: Hematoxylin and eosin
IL-1β: Interleukin-1 beta
LM: Light micrographs
MDA: Malondialdehyde
NPs: Nanoparticles
NIOSH: National Institute for Occupational Safety and Health
NADPH: Nicotinamide adenine dinucleotide phosphate
SOD: Superoxide dismutase
TiO_2_: Titanium dioxide
TiO_2_NPs: Titanium dioxide nanoparticles
TEM: Transmission electron microscopy
TNF-α: Tumor necrosis factor alpha
ROS: Reactive oxygen species
ZnO: Zinc oxide
