# Supplementary figures and images for: The toxic effect of titanium dioxide nanoparticles on rat submandibular salivary glands and the protective role of vitamin E
Source: BMC Oral Health. 2025 Aug 20;25:1342. doi: 10.1186/s12903-025-06631-w (PMC12366081; doi:10.1186/s12903-025-06631-w)

## Slide 1
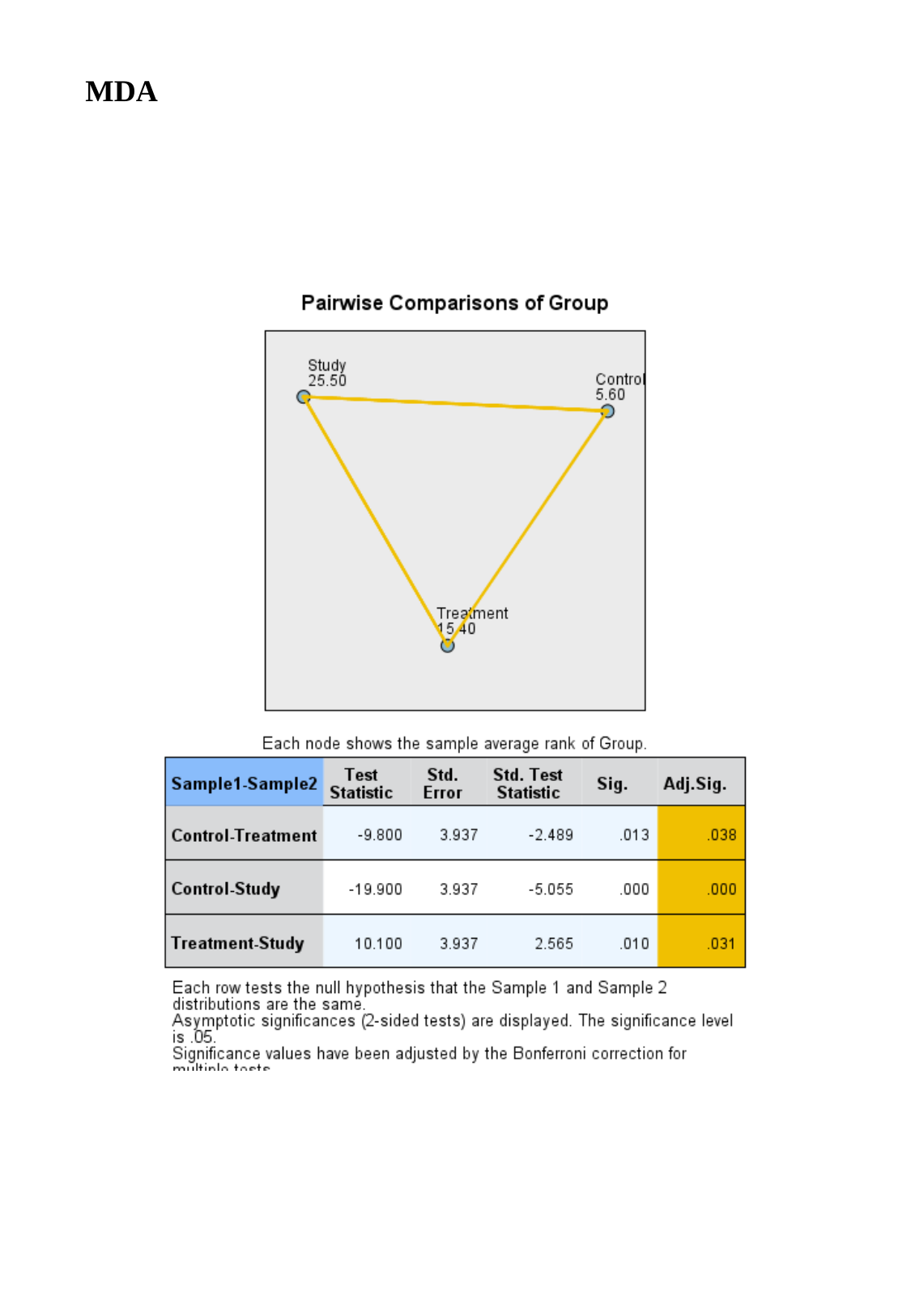

MDA

## Slide 2
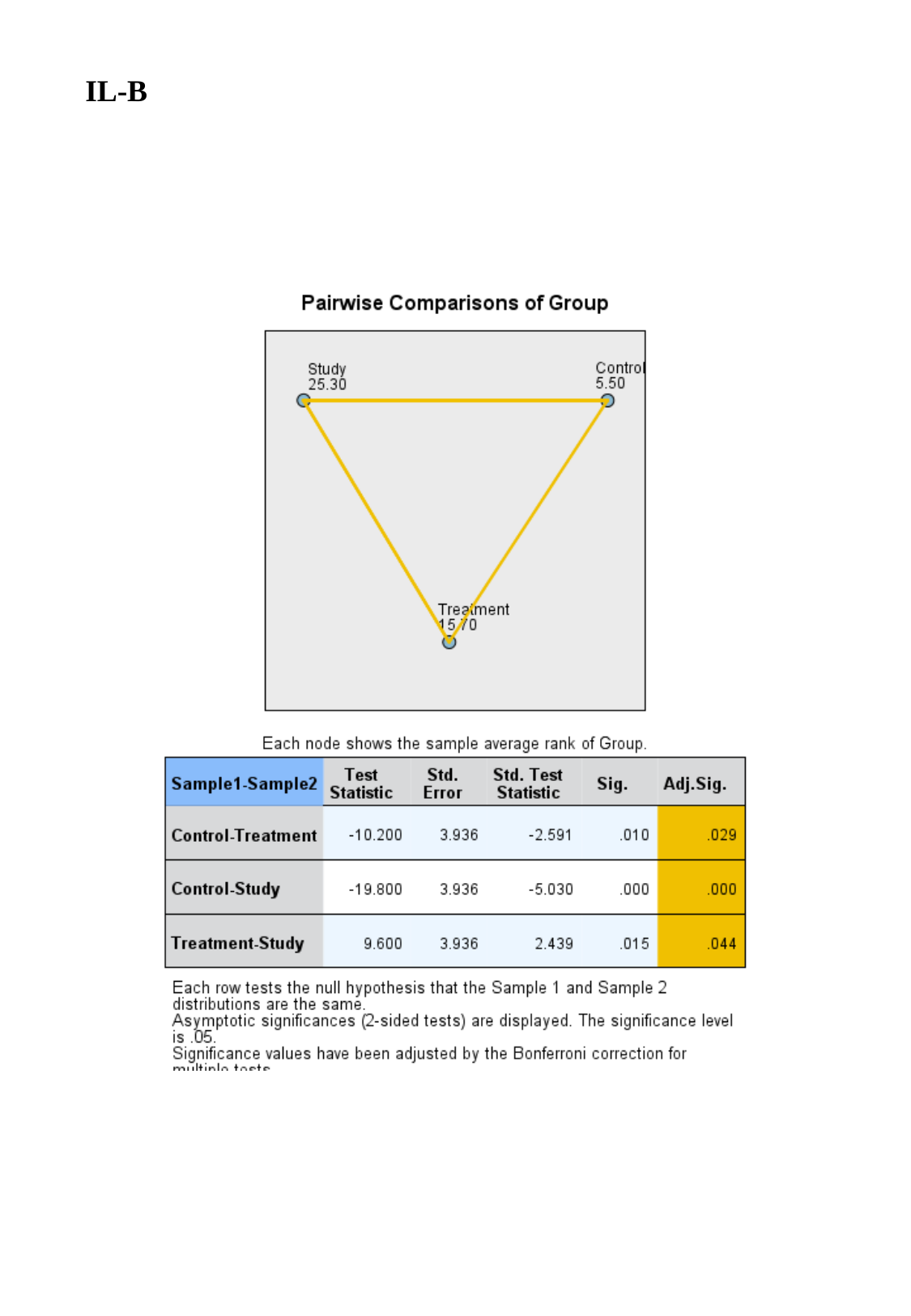

IL-B

## Slide 3
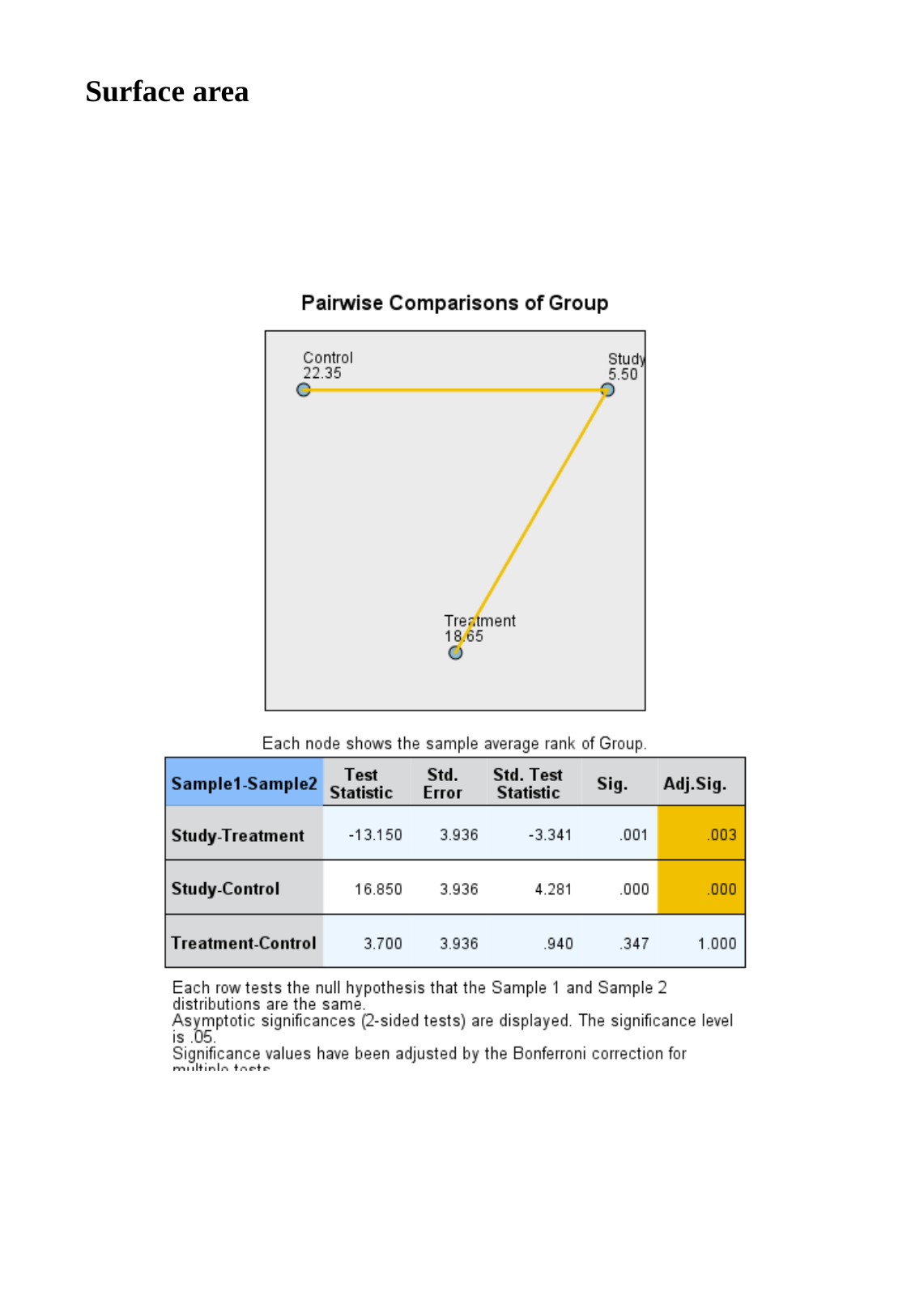

Surface area

Supplement: Supplementary file 1 — Supplementary Material 1 [file 12903_2025_6631_MOESM1_ESM.pptx]
