# Supplementary material for: The toxic effect of titanium dioxide nanoparticles on rat submandibular salivary glands and the protective role of vitamin E
Source: BMC Oral Health. 2025 Aug 20;25:1342. doi: 10.1186/s12903-025-06631-w (PMC12366081; doi:10.1186/s12903-025-06631-w)
Supplement: Supplementary file 2 — Supplementary Material 2 [file 12903_2025_6631_MOESM2_ESM.pptx]

## Slide 1
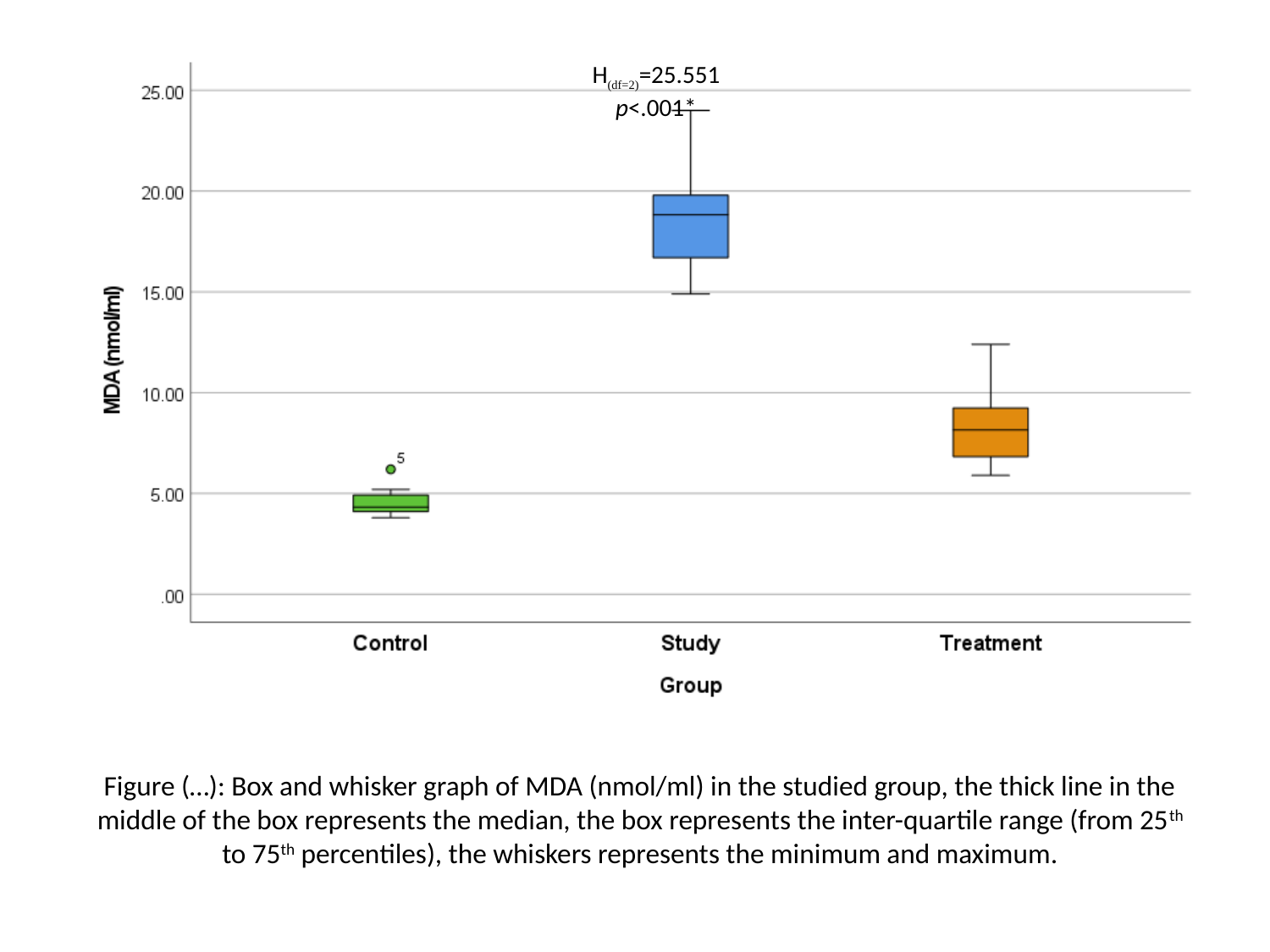

H(df=2)=25.551
p<.001*
Figure (…): Box and whisker graph of MDA (nmol/ml) in the studied group, the thick line in the middle of the box represents the median, the box represents the inter-quartile range (from 25th to 75th percentiles), the whiskers represents the minimum and maximum.

## Slide 2
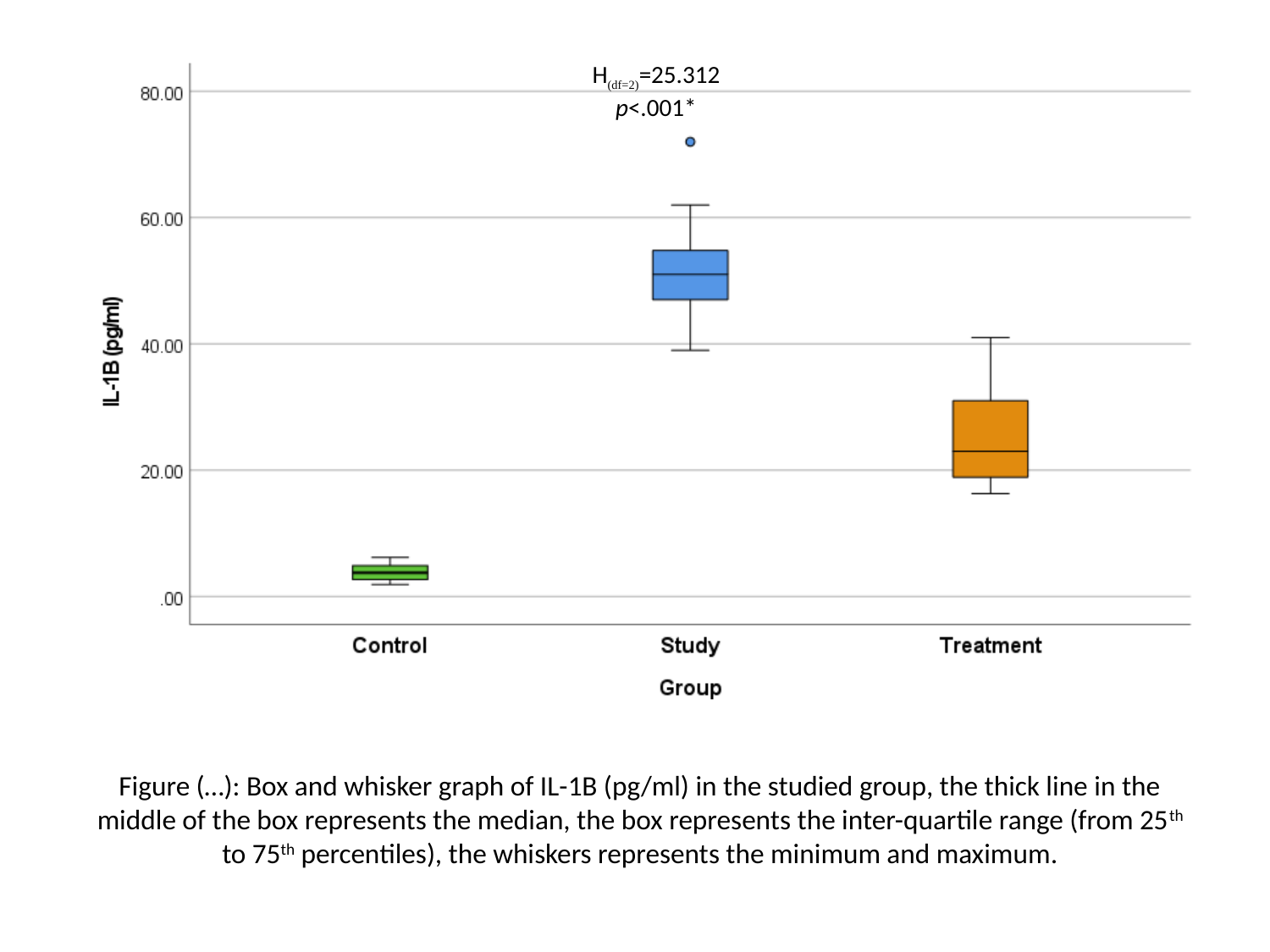

H(df=2)=25.312
p<.001*
Figure (…): Box and whisker graph of IL-1B (pg/ml) in the studied group, the thick line in the middle of the box represents the median, the box represents the inter-quartile range (from 25th to 75th percentiles), the whiskers represents the minimum and maximum.

## Slide 3
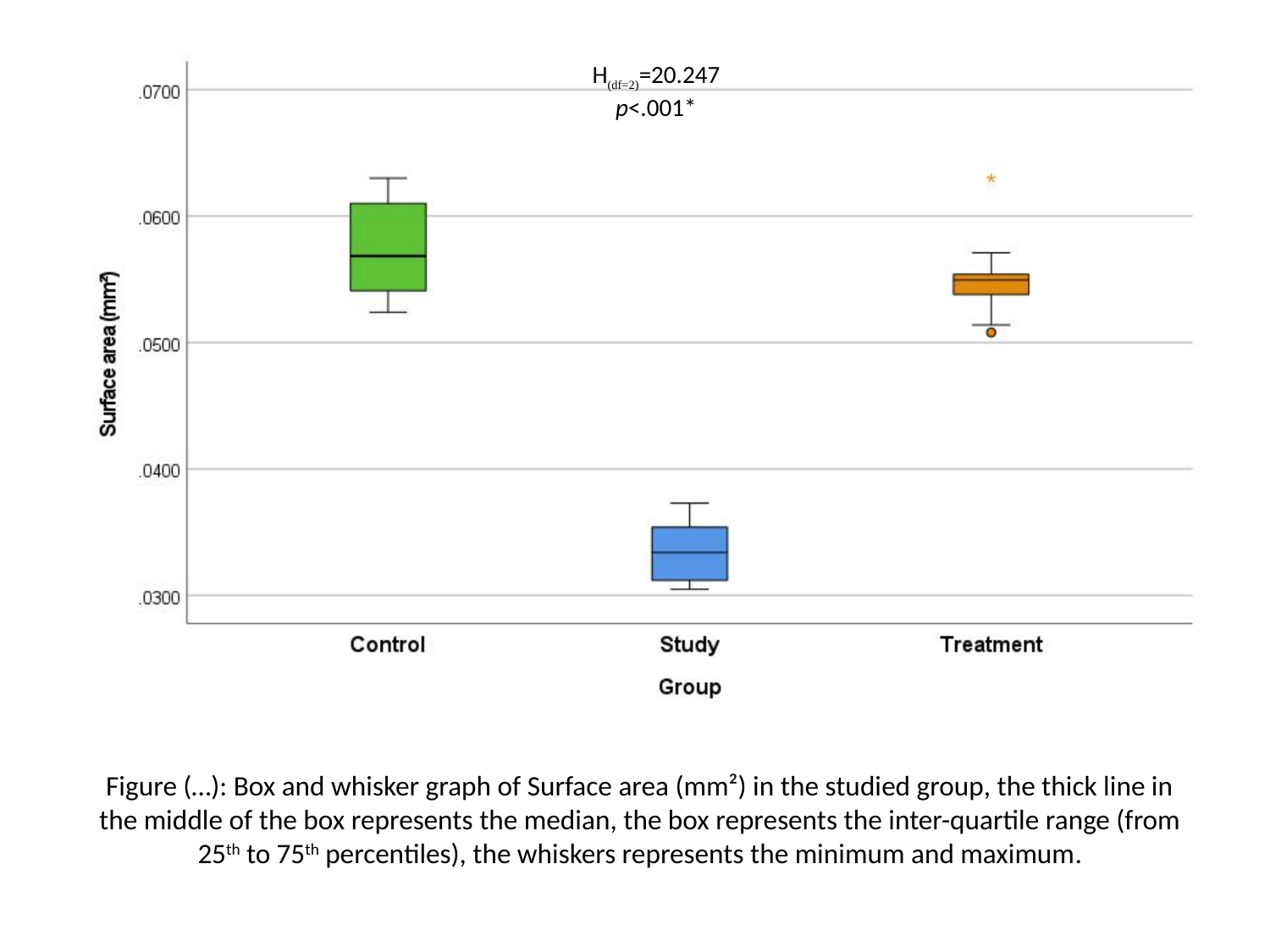

H(df=2)=20.247
p<.001*
Figure (…): Box and whisker graph of Surface area (mm²) in the studied group, the thick line in the middle of the box represents the median, the box represents the inter-quartile range (from 25th to 75th percentiles), the whiskers represents the minimum and maximum.
